# Advances and Challenges in KRAS Mutation Detection and Clinical Implications

**DOI:** 10.3390/cancers18010031

**Published:** 2025-12-22

**Authors:** Maryam Sadat Mirlohi, Tooba Yousefi, Javad Razaviyan, Samira Nomiri, Esmail Pishbin, Meer-Taher Shabani-Rad, Mohammad Reza Ahmadian, Siamak Salami

**Affiliations:** 1 Department of Clinical Biochemistry, School of Medicine, Shahid Beheshti University of Medical Sciences, Tehran 1985717413, Iran; maryammirlohi@uvic.ca (M.S.M.); toobayousefi@sbmu.ac.ir (T.Y.); razaviyan@rayana.ir (J.R.); s.nomiri@sbmu.ac.ir (S.N.); 2 Department of Mechanical Engineering, University of Victoria, Victoria, BC V8W 2Y2, Canada; 3 Bio-Microfluidics Laboratory, Department of Electrical Engineering and Information Technology, Iranian Research Organization for Science and Technology, Tehran 3313193685, Iran; pishbin@irost.org; 4 Department of Pathology & Laboratory Medicine, University of Calgary/Alberta Precision Laboratories (APL), Calgary, AB T2N 1N4, Canada; meer-taher.shabani-rad@cls.ab.ca; 5 Institute of Biochemistry, Molecular Biology Medical Faculty, Heinrich-Heine University, 40225 Düsseldorf, Germany; reza.ahmadian@hhu.de

**Keywords:** KRAS mutations, precision medicine, oncogene, molecular method, targeted therapy

## Abstract

Aberrant activation of the RAS signaling pathway is a halmark of various cancers. This activation, is often caused by mutations in RAS genes or other pathway components and, drivesi uncontrolled cell growth and proliferation. Studies have demonstrated that certain codon mutations can significantly influence the clinical outcomes of cancer patients. Historically, KRAS was considered “undruggable”; however, recent advancements in drug discovery have led to the development of promising KRAS inhibitors. Accurately identifying the specific type of KRAS mutation in a patient is essential for making optimal treatment decisions. Several methods have been developed for detecting KRAS mutations to address this need, focusing on creating robust, rapid, sensitive, accurate, and cost-effective approaches, particularly for point-of-care applications. Starting with the Ras family and RASopathies, this review provides a comprehensive overview of KRAS mutation detection methods, ranging from research-use-only techniques to in vitro diagnostic-certified tests. Published results are critically evaluated in terms of accuracy, sensitivity, cost, throughput, and suitability for various sample types and clinical settings. This, offers researchers and clinicians an up-to-date resource for.

## Simple Summary

Mutations in the RAS signaling pathway, especially in the KRAS oncogene, are major drivers of cancer progression, therapy resistance, and poor clinical outcomes. For decades KRAS was considered undruggable, but recent advances have produced selective KRAS inhibitors, creating new opportunities for personalized cancer treatment. Accurate identification of KRAS mutations is therefore essential for selecting targeted therapies, predicting drug response, and guiding clinical decisions. This review provides an overview of established and emerging KRAS detection methods, comparing their sensitivity, specificity, cost, and clinical applicability. The goal is to support researchers and clinicians in improving cancer diagnostics and optimizing patient care.

## 1. Personalized Cancer Treatment: The Role of Mutation Screening

Precision medicine seeks to tailor treatment based on the specific genetic and biological characteristics of each patient. In oncology, this approach relies heavily on detecting driver mutations such as KRAS, one of the most frequently altered oncogenes in solid tumors, to guide therapy selection. Mutation screening is therefore central to precision oncology, enabling clinicians to identify actionable mutations, match patients with targeted drugs, avoid ineffective therapies, and improve outcomes [1,2,3]. Recent advances in diagnostic technologies now allow the rapid and accurate detection of cancer-related mutations in both tissue and liquid biopsies, making it feasible to incorporate molecular profiling into routine practice. Such progress has led to measurable improvements in patient management, with enhanced survival and reduced toxicity compared with conventional chemotherapy [4,5,6].

In this review, KRAS mutation detection is emphasized as a crucial part of precision medicine. Our review compares methods based on sample type, platform, sensitivity, specificity, limit of detection, workflow, turnaround time, throughput, and cost. Our study also examines how these technical features affect clinical implications, including diagnosis, prognosis, treatment decisions, drug resistance, and clinical trial eligibility. RAS background is kept brief in this section, with detailed analysis left to later sections, where KRAS-driven cancer detection technologies are critically evaluated.

## 2. RAS Family of Small GTPases

Rat Sarcoma virus (RAS) genes encode four proteins derived from small guanosine triphosphatase (GTPase) binding proteins with an 82–90% sequence identity [7]. These proteins function as molecular switches that, cycle between an inactive Guanosine Diphosphate or GDP-bound and an active GTP-bound states. This progress regulates signaling downstream of many growth factor receptors, such as epidermal growth factor receptors (EGFRs), Hepatocyte Growth Factor (HGF) receptors (MET), or SCF receptors (KIT) [8]. There are three major members of this family: HRAS, NRAS, and KRAS, which are structurally similar but differ in tissue distribution and role in cancer [9,10].

Despite their similarities as signaling molecules, they play different roles in cancer and responsd differently to treatment. This highlights the importance of understanding these differences when developing targeted cancer therapies [11,12].

## 3. RAS Family Signaling Pathways

The RAS proteins transduce extracellular signals from receptor tyrosine kinases (RTKs) to intracellular pathways that regulate proliferation, differentiation, and survival [13,14]. Upon binding of ligands to RTKs, guanine nucleotide exchange factors (GEFs) stabilize the nucleotide-free state, allowing GDP release and subsequent GTP binding. A conformational change activates RAS and allows it to interact with downstream effectors, most notably the RAF–MEK–ERK cascade, which regulates proliferation and differentiation, as well as the PI3K–AKT–mTOR pathway, which regulates cell growth and survival, and the RALGDS pathway, which promotes migration and invasion [15,16].

A GTPase-activating protein (GAP) stimulates GTP hydrolysis, and a negative feedback loop terminates pathway activity to maintain cellular homeostasis through signal termination. KRAS mutations lead to persistent signaling, uncontrolled cell division, and tumor progression, emphasizing the need for precise detection strategies and targeted therapeutics [9,17].

## 4. RASopathies

RASopathies are a group of developmental syndromes caused by germline mutations in genes encoding components of the RAS–MAPK signaling pathway. These alterations produce overlapping clinical features, including congenital heart defects, distinctive craniofacial dysmorphisms, neurocognitive impairment, and, in some cases, tumor predisposition. While KRAS mutations are relatively rare, they have been reported in subsets of Noonan and cardiofaciocutaneous syndromes, underscoring the systemic impact of aberrant RAS signaling beyond cancer. This section is included to provide broader biological context, while the central focus of the review remains on somatic KRAS mutations in oncology [18,19,20]. A concise overview of the major RASopathies [21,22,23,24,25,26,27,28], their causative genes, and hallmark clinical features is provided in Table 1.

## 5. KRAS: A Historical Timeline

KRAS (Kirsten rat sarcoma viral oncogene homolog) is found on chromosome 12p12.1. It consists of six exons that encode a 21 kDa small GTP-binding protein [29,30]. In 1982, it was discovered that human RAS is a homolog of viral RAS oncogenes, establishing its importance in cancer biology. Soon after, KRAS was identified as a molecular switch protein, cycling between inactive GDP- and active GTP-bound states [31,32].

Two isoforms of KRAS were discovered in 1989, KRAS4A and KRAS4B. Both share the same G-domain but differ in their hypervariable C-terminal regions, which regulate post-translational modifications and subcellular localization [33,34]. The KRAS4B gene is expressed in most adult tissues and cancer cells [32].

In the 1990s, KRAS mutations were clearly linked to pancreatic (90%) and colorectal cancer (40–50%), emphasizing their role as an oncogenic motor [35,36]. Those discoveries led to the integration of KRAS mutation analysis into cancer diagnostics and therapeutics. In the 2000s, advances in sequencing technologies enabled the clinical application of KRAS mutation detection, particularly to predict resistance to anti-EGFR therapy [37]. Recently, the development of KRAS G12C inhibitors (e.g., sotorasib, adagrasib) has paved the way for major breakthroughs in the treatment of KRAS-driven cancers [38,39].

**Figure 1:**
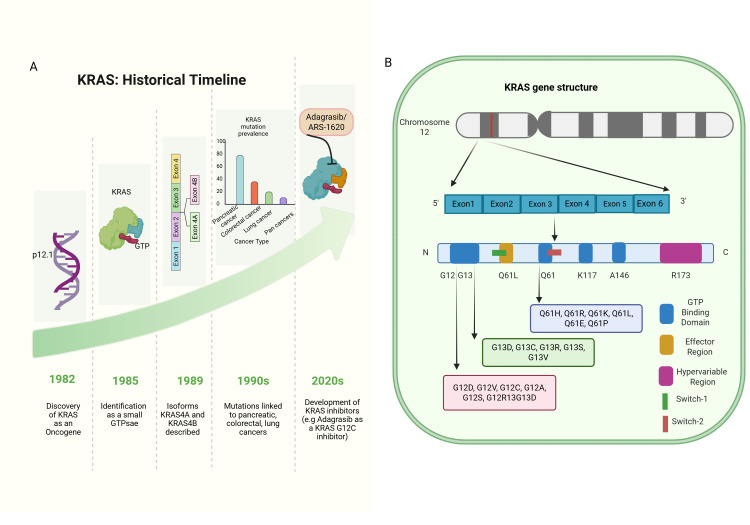
Spatial distribution of KRAS mutation hotspots and incidence rates; KRAS timeline and gene structure. (**A**) Milestones in KRAS research, including the discovery of RAS oncogenes, identification of KRAS isoforms, clinical adoption of KRAS mutation testing, and the development of targeted KRAS G12C inhibitors. The timeline now includes *ARS-1620*, the first KRAS G12C inhibitor developed in preclinical studies, followed by *sotorasib* (AMG-510), the first FDA-approved KRAS inhibitor, and *adagrasib* (MRTX849), which later demonstrated clinical efficacy. (**B**) Genomic organization of the KRAS gene, illustrating exon structure and major pathogenic mutation hotspots, with codons 12, 13, and 61 accounting for ~85% of KRAS alterations across cancers. Created in BioRender (2025): https://BioRender.com/7wu8nnt.

The timeline in Figure 1A illustrates the discovery, structural insights, oncogenic associations, clinical adoption of detection, and emergence of targeted inhibitors.

KRAS functions as a small GTPase molecular switch cycling between inactive GDP- and active GTP-bound states, regulated by GEFs and GAPs [40,41]. Activated KRAS transduces signals primarily through key effector pathways at the plasma membrane. Proliferation and differentiation are governed by the RAF–MEK–ERK pathway, survival and metabolism by the PI3K–AKT–mTOR pathway, and migration and invasion by the RalGDS pathway. A negative feedback mechanism such as ERK-dependent inhibition of upstream receptors ensures that signaling is controlled under normal circumstances [42,43].

Oncogenic KRAS mutations disrupt this regulatory balance, producing a constitutively active protein that continuously stimulates downstream pathways. The metabolic reprogramming caused by such mutations includes enhanced glucose uptake, shift from oxidative phosphorylation to aerobic glycolysis (the Warburg effect), and increased lactate production. Furthermore, mutant KRAS contributes to tumor progression by secreting inflammatory cytokines such as GM-CSF and IL-6, which recruit immune and stromal cells. By cross-talking with cancer-associated fibroblasts, KRAS-mutant tumor cells promote extracellular matrix remodeling (collagen, hyaluronic acid), which enhances tumor growth and invasion [32,44].

**Figure 2:**
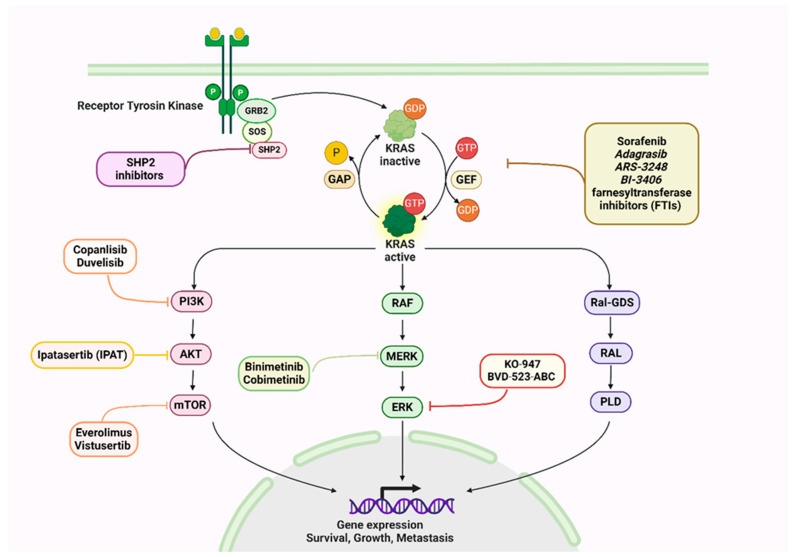
Overview of the KRAS signaling pathway highlighting major downstream cascades (RAF–MEK–ERK, PI3K–AKT–mTOR, RalGDS) and the sites targeted by current inhibitors, including G12C covalent blockers (sotorasib, adagrasib) and upstream modulators (SHP2 and SOS1 inhibitors). Created in BioRender (2025): https://BioRender.com/qgwxh8b.

Many cancers are characterized by the dysregulation of KRAS signaling. KRAS has been identified as a central oncogenic driver, resulting in decades of research aimed at pharmacologically targeting this pathway, though effective KRAS inhibitors have only recently been developed [45]. As illustrated in Figure 2, oncogenic KRAS activates multiple signaling branches, including RAF–MEK–ERK and PI3K–AKT–mTOR, which collectively drive proliferation and survival. This figure also highlights where recently developed inhibitors act within the pathway, clarifying the mechanistic rationale for targeted therapies.

## 6. KRAS Inhibitors and KRAS-Targeted Therapies

Targeting KRAS has historically posed a significant challenge due to its picomolar affinity for guanine nucleotides and the lack of deep allosteric pockets suitable for small-molecule binding. Early attempts, including farnesyltransferase inhibitors (FTIs), failed in KRAS-driven cancers owing to compensatory alternative prenylation, reinforcing its reputation as “undruggable.” However, advances in structural biology and biochemical analyses uncovered mutation-specific vulnerabilities, particularly in the KRAS G12C mutant. This insight facilitated the development of covalent inhibitors that selectively trap KRAS G12C in an inactive, GDP-bound conformation [46]. The first clinical breakthrough was achieved with sotorasib (AMG 510), an irreversible covalent inhibitor targeting KRAS G12C, which received FDA approval for advanced non–small cell lung cancer (NSCLC) [47,48].

Following this, adagrasib (MRTX849), another covalent G12C inhibitor with favorable pharmacokinetic properties, demonstrated efficacy in NSCLC and colorectal cancers [49,50,51]. Several other compounds, such as ARS-3248, are currently in early clinical development, showing promising preclinical activity [52]. Targeting upstream regulators of KRAS activation represents a complementary approach. BI-3406, an oral SOS1 inhibitor, impedes nucleotide exchange and reduces KRAS-GTP loading, especially when combined with MEK inhibitors [53,53]. Similarly, SHP2 inhibitors block a critical phosphatase that transduces receptor tyrosine kinase signals to RAS and exhibit synergy with KRAS G12C inhibitors [54]. Indirect and broad-spectrum strategies include pan-RAS inhibitors, targeted protein degraders, engineered toxins, and RNA-based therapies such as siRNAs and cancer vaccines. While mostly in preclinical stages, these approaches diversify the therapeutic landscape [46]. Notably, although initial FTI treatments in KRAS-mutant cancers were unsuccessful, renewed interest in selective FTIs and rational combination therapies suggests potential utility in specific contexts [55,56]. Details of the currently available KRAS inhibitors and their classification are summarized in Figure 2.

Despite the clinical success of KRAS G12C inhibitors, intrinsic and acquired resistance have emerged as major therapeutic challenges. Recent studies have shown that resistance arises through multiple mechanisms. Upregulation of SHP2, SOS1, AURKA, and YAP/TAZ signaling has also been implicated in promoting drug escape. Building on these insights, several combinatorial strategies have been proposed to enhance therapeutic efficacy and delay resistance. These include dual KRAS + SHP2 inhibition, combined blockade of RTK/RAS feedback circuits, and MEK or ERK inhibitors used synergistically with KRAS G12C inhibitors. Additionally, emerging data support combining KRAS-targeted agents with immune checkpoint inhibitors, particularly in tumors exhibiting adaptive immunosuppression following KRAS inhibition [57,58].

Collectively, these advances represent a paradigm shift in KRAS targeting. Nevertheless, the emergence of adaptive resistance and limited durability of responses underscores the necessity for combination strategies integrating KRAS inhibitors with agents targeting SHP2, SOS1, or downstream effectors to enhance therapeutic efficacy. Details of the main therapeutic strategies are summarized in Table 2.

## 7. The Importance of KRAS Mutation Detection

The detection of KRAS mutations, among the most frequent genetic alterations in human cancers such as colorectal, pancreatic, and lung adenocarcinomas, constitutes a cornerstone of molecular oncology. Accurate identification of these mutations plays a vital role in multiple clinical contexts. Firstly, KRAS mutation profiling aids in diagnosis and tumor classification, given the association of specific mutations with distinct cancer types [32,63]. Secondly, mutation status holds prognostic value, with certain KRAS mutants correlating with adverse outcomes; for instance, colorectal cancer patients harboring KRAS mutations typically exhibit a poorer prognosis than their wild-type counterparts [64,65,66]. Importantly, KRAS mutation testing informs therapeutic decision-making, as the efficacy of anti-EGFR agents like cetuximab and panitumumab is limited to patients with KRAS wild-type tumors, while targeted inhibitors such as sotorasib and adagrasib specifically benefit those with KRAS G12C mutations [67,68].

Lastly, precise mutation detection is indispensable for patient selection in clinical trials, ensuring optimized evaluation of emerging therapies and personalized treatment strategies [38,69]. These multifaceted clinical implications underscore the necessity for robust and sensitive KRAS mutation assays to guide effective patient management.

## 8. Hot Spots for KRAS Mutations

KRAS mutations predominantly occur within the GTPase domain, which plays a crucial role in downstream signaling. Among the four coding exons, exon 2 exhibits the highest mutation frequency, impairing GTP hydrolysis and resulting in constitutively active KRAS proteins [70]. The vast majority of mutations cluster at hotspot codons 12, 13, and 61, accounting for about 85% of KRAS alterations in human cancers [71]. Figure 1B illustrates a map depicting the locations with elevated occurrences of KRAS mutations, known as hot spots, and the corresponding incidence rates. Codon 12 mutations are the most prevalent, especially substitutions of glycine for aspartic acid (G12D), valine (G12V), or cysteine (G12C). G12D represents approximately 50% of all KRAS mutations and is commonly encountered in colorectal, pancreatic, and lung adenocarcinomas. G12V accounts for around 25%, although also prevalent in these cancers. The G12C mutation, though less common overall (~5%), is notably frequent in lung adenocarcinoma and is therapeutically actionable with novel inhibitors like sotorasib and adagrasib [45,72,73]. Mutations at codon 13, primarily G13D, constitute 15–25% of KRAS mutations and share similar cancer distributions, including colorectal, pancreatic, and lung cancers. Less frequent codon 13 variants include G13C, G13R, and G13S, with some differences in tumor type prevalence [45,72,74]. Codon 61 mutations are rare but have been identified in various cancers such as thyroid carcinoma and melanoma. Common substitutions include Q61R and Q61K, with less frequent variants like Q61H, Q61L, and Q61E also reported [73]. Though less common, these mutations contribute to oncogenic activation and may influence treatment responses.

Clinically, the mutation spectrum impacts prognosis and therapeutic strategies. For example, codon 12 mutations occur in 40–50% of colorectal cancers and dominate pancreatic tumors (~90%), while codon 13 mutations are less frequent (5–10%). In lung cancers, KRAS mutations affect roughly 25% of non-small cell lung cancers, with codon 12 mutations predominant. Similar mutation distributions are observed in the biliary tract and ovarian cancers [45,75].

Understanding the specific KRAS mutation type is critical, as it shapes oncogenic signaling properties, therapeutic vulnerabilities, and resistance profiles. This informs precision oncology approaches.

## 9. Detection Methods of KRAS Mutations

**Figure 3:**
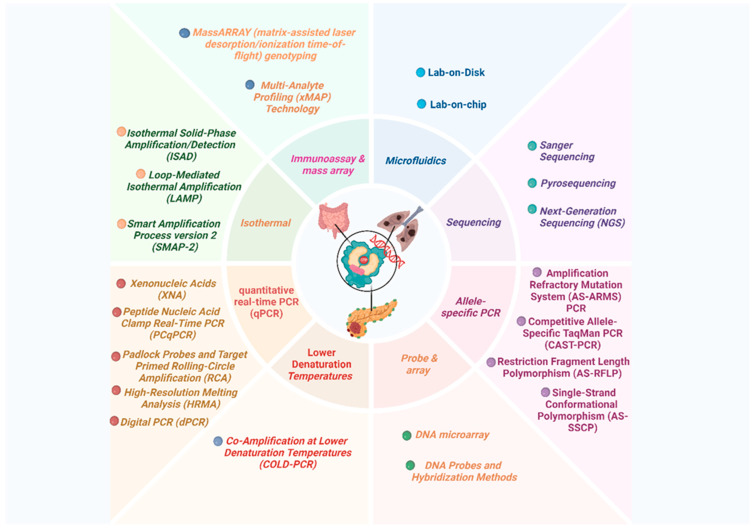
Overview of KRAS mutation detection platforms and their clinical utility. Schematic representation of major methodologies used for KRAS genotyping, including Sanger sequencing, pyrosequencing, allele-specific PCR, digital PCR, NGS, and emerging microfluidic and isothermal amplification technologies. This figure summarizes key analytical differences such as sensitivity, mutation coverage, turnaround time, and suitability for tissue versus liquid biopsy samples. Methods positioned toward the right of the diagram demonstrate higher sensitivity and clinical applicability for minimal residual disease monitoring and real-time treatment response assessment. Created in BioRender. (2025) https://BioRender.com/2t8l9fq.

A diverse array of molecular techniques is available for KRAS mutation detection, each offering distinct analytical performance characteristics and varying clinical utility. Selection of the optimal method depends on critical factors such as mutation detection sensitivity, sample compatibility, assay throughput, and compliance with regulatory standards. The increasing recognition of biological and clinical heterogeneity in KRAS-driven cancers, both across tissue types and among mutation subtypes, necessitates the use of highly sensitive, multiplexed, and rigorously validated assays capable of supporting precision oncology applications, including therapy stratification and real-time monitoring of resistance [76,77,78]. To aid interpretation, Figure 3 organizes detection methods according to their analytical performance and clinical maturity. Traditional sequencing approaches provide broad coverage but limited sensitivity, whereas allele-specific PCR and digital PCR enable highly sensitive hotspot detection, particularly in cfDNA. Emerging microfluidic and isothermal platforms offer rapid, point-of-care potential but remain largely research-use. This visual comparison helps to clarify why testing strategies differ across clinical contexts.

KRAS mutations can be detected in a variety of clinical specimens, including formalin-fixed paraffin-embedded (FFPE) tissue, biopsy or cytology samples, fine-needle aspirates (FNAs), and liquid biopsy materials such as blood, plasma, and urine [79,80,81]. In endoscopic ultrasound-guided fine-needle aspiration (EUS-FNA) samples, KRAS mutation analysis has proven highly effective for distinguishing malignant from benign lesions, improving diagnostic accuracy in pancreatic and other tumors [82,83].

In recent years, liquid biopsy approaches have gained major clinical and research interest, since circulating tumor DNA (ctDNA), circulating tumor cells (CTCs), and other analytes reflect both primary tumors and metastatic burden [84]. Detection of KRAS mutations in plasma or serum is often associated with poor prognosis and disease progression, highlighting its potential for real-time monitoring [32].

Several studies have demonstrated that cfDNA-based KRAS testing offers strong analytical performance. For example, in a cohort of 106 patients with primary and metastatic colorectal cancer, cfDNA analysis achieved 98% specificity and 92% sensitivity across seven KRAS point mutations, with a concordance rate of 96% compared with matched tissue samples [85,86,87]. These findings underscore the reliability of liquid biopsy as a complement to tissue testing. Furthermore, advances in lab-on-a-chip microdevices are accelerating the translation of cfDNA assays into routine oncology practice [88].

Classical Sanger sequencing has historically served as the foundational approach for KRAS mutation identification, providing comprehensive exon coverage and enabling the detection of both common and rare variants. However, it necessitates relatively high mutant allele frequencies (≥10–20%) and is thus limited in utility for low-purity tissue specimens and plasma-derived circulating tumor DNA (ctDNA) analyses. Recent methodological enhancements involving capillary electrophoresis have marginally advanced sensitivity, yet limitations regarding throughput and cost persist [89,90,91,92,93,94]. Pyrosequencing offers improved sample throughput and quantitative evaluation of hotspot mutations, boasting limits of detection (LOD) typically between 2 and 5%. It is widely implemented on solid tissue and liquid biopsy specimens, including cytology and plasma cell-free DNA (cfDNA). This method remains constrained by pre-defined assay designs and susceptibility to sequence-dependent errors [95,96,97,98,99,100,101,102].

Next-generation sequencing (NGS) platforms now represent the most comprehensive platforms for KRAS mutation analysis, extending coverage across clinically relevant KRAS exons and codons while concurrently detecting co-occurring alterations that inform tumor biology and therapeutic responsiveness. NGS is indispensable for broad genomic profiling, enrolling patients in clinical trials, and dissecting resistance mechanisms. Despite these advantages, NGS remains resource intensive, costly, and characterized by longer turnaround times than targeted assays [103,104,105,106,107,108,109,110,111]. Allele-specific PCR (AS-PCR) methods—encompassing amplification refractory mutation system PCR (ARMS-PCR), competitive allele-specific TaqMan PCR (CAST-PCR), and Scorpion probe technologies—enable the rapid and highly sensitive detection of actionable KRAS variants (LOD < 1%). These approaches underpin numerous Food and Drug Administration (FDA)- and CE-In Vitro Diagnostic (IVD)-approved companion diagnostics, predominantly applied in colorectal and non-small cell lung cancers. However, their utility is limited to predetermined hotspot mutations, precluding identification of novel variants [112,113,114,115,116,117,118,119,120,121,122,123,124,125,126,127,128].

Digital PCR (dPCR) technologies, including droplet digital PCR (ddPCR) and chamber digital PCR (cdPCR), have transformed non-invasive KRAS testing by facilitating absolute quantification of allele fractions with analytic sensitivities down to 0.01%. This capability is particularly valuable for ctDNA applications such as minimal residual disease (MRD) monitoring and longitudinal assessment of therapeutic response. These modalities, however, remain constrained by their targeted design scope and the need for specialized instrumentation [129,130,131,132,133].

Pre-amplification enrichment strategies such as co-amplification at lower denaturation temperature PCR (COLD-PCR) [134,135,136,137] and clamp-based chemistries employing peptide nucleic acids (PNA) or xenonucleic acids (XNA) further augment minority allele detection by selectively suppressing wild-type amplification, thereby enhancing assay sensitivity [138,139,140,141,142,143,144,145,146,147].

Additional emerging methods include loop-mediated isothermal amplification (LAMP), matrix-assisted laser desorption/ionization time-of-flight mass spectrometry (MALDI-TOF MS) using the MassARRAY® System (Agena Bioscience, San Diego, CA, USA) [148,149,150,151], and DNA microarray/xMAP multiplex platforms [148,152,153]. These methodologies offer advantages related to rapid turnaround, automation, and scalability; however, the majority remain classified as research-use only (RUO) or are in the nascent stages of clinical validation. Likewise, biosensor-based assays, rolling circle amplification (RCA) with padlock probes [154,155,156,157], and microfluidic lab-on-chip platforms are expanding the KRAS mutation detection toolbox, facilitating rapid and ultra-sensitive analyses. For instance, integration of microfluidics in cfDNA analysis demonstrates promising potential for routine oncologic workflows and point-of-care molecular diagnostics [158,159,160,161].

Among emerging platforms, a recently reported fluorescence-based long block displacement amplification (LBDA) method exemplifies a significant advance in KRAS genotyping. LBDA achieved a remarkably low limit of detection (0.08% variant allele frequency) while simultaneously targeting 81 mutation hotspots. Clinical validation in colorectal cancer tissue samples demonstrated high sensitivity (88%) and specificity (100%) compared with NGS, but with reduced assay time and cost, underscoring its translational potential [162].

Table 3 provides a comparative summary of these KRAS detection methodologies with insights into analytical sensitivity, mutation coverage, throughput, cost, and regulatory status. This comprehensive overview emphasizes trade-offs between established and innovative techniques, highlighting the relevance of each for specific KRAS mutation testing scenarios in clinical oncology.

## 10. Technical and Clinical Comparison of KRAS Mutation Detection Techniques

KRAS mutation detection technologies span a wide technical spectrum, each exhibiting distinct analytical performances, clinical utilities, and operational constraints. While foundational methods like Sanger sequencing laid the groundwork for KRAS analysis, their clinical utility has dwindled in the precision medicine era due to inherent limitations in their sensitivity, particularly in mixed tumor samples or degraded specimens [90,182,183,184]. Studies highlight the Sanger method’s failure to detect mutant alleles below approximately 10–20%, which compromises treatment decisions, especially in low-purity FFPE tissues commonly encountered in clinical oncology [183]. Pyrosequencing improves sensitivity to 2–5% mutant allele frequency with quantitative outputs, but remains limited by predefined hotspot panels and susceptibility to homopolymeric sequencing errors, confining its utility predominantly to common KRAS mutations [95,96,97,98,185]. NGS provides a comprehensive mutation profile, covering exons 2, 3, and 4 with high multiplexing capacity, enabling co-mutation detection and broad genomic characterization vital for clinical trial stratification. However, real-world experience with NGS reveals substantive failure rates, especially from FFPE samples characterized by DNA fragmentation and chemical modifications, which challenge assay robustness and introduce false negatives or reporting delays [104,105,106,107,111,183]. These failure rates bear direct clinical consequences due to delayed therapy initiation or misclassification affecting eligibility for targeted drugs or trials.

Bolton et al. further demonstrated strong concordance between CastPCR and Therascreen in FFPE colorectal cancer, confirming that CastPCR offers reproducible and clinically reliable results, with only minor technical differences in Ct values that are not clinically significant [186]. Suzuki et al. reported an 81.4% concordance between nested PCR-based sequencing and QProbe methods in colon cancer, highlighting how assay-specific variation can still yield broadly comparable outcomes when quality controls are enforced [187]. Matsunaga et al. compared direct sequencing, Scorpion-ARMS, pyrosequencing, and Luminex xMAP, and concluded that all three sensitive assays achieved high concordance, reinforcing their suitability for KRAS detection in CRC [188].

Comparative benchmarking exemplifies the variability in platform performance. Sherwood et al.’s multi-center evaluation demonstrated a broad call accuracy span (55.8–96%) across 13 mutation detection platforms, with superior accuracy from automated qPCR systems such as Idylla, digital PCR (ddPCR), and MassARRAY (UltraSEEK), while standard sequencing and some NGS panels lagged behind [183]. These findings corroborate concerns about conventional sequencing insufficiency and urge adoption of more sensitive, automated platforms where feasible. Recent studies further reinforce these conclusions: Szeto et al. directly compared ddPCR with NGS for ctDNA in rectal cancer patients and reported that ddPCR outperformed NGS in sensitivity for low-frequency alleles, underscoring ddPCR’s clinical value in liquid biopsy settings [189]. Similarly, Murakami et al. showed that while NGS panels provided broader mutational context, PCR-based approaches were faster and less failure-prone in routine diagnostic workflows [190].

Further, Gao et al. confirmed superior sensitivity of the KRAS StripAssay relative to Sanger and pyrosequencing in FFPE colorectal cancer tissue, detecting mutations at lower allele frequencies and thus increasing mutation detection rates critical for effective therapy and trial inclusion [185]. Chretien et al. observed low but non-negligible discordance (~2%) between HRM, TaqMan PCR, and PCR-RFLP, underscoring the benefit of orthogonal or confirmatory testing to resolve borderline or ambiguous results, especially in heterogeneous samples [191]. Franklin et al. and Ibrahem et al. supported the enhanced sensitivity of PCR-based methods over Sanger, albeit each confers unique trade-offs in cost, throughput, and workflow complexity [90,184]. Lee et al. expanded this evidence to cytology specimens, showing that pyrosequencing achieves mutation detection rates equivalent to histology, thereby validating cytology as a practical alternative specimen type in lung cancer diagnostics [192].

Emerging isothermal and microfluidic platforms further broaden the clinical toolbox. Jin et al. demonstrated that the ISAD assay detects KRAS G12D/G13D mutations in CRC within 30 min, with a detection limit of just 1% mutant alleles, outperforming PCR-based and direct sequencing approaches for rapid clinical decisions [193]. Tanaka et al. enhanced dPCR resolution by integrating melting curve analysis, enabling discrimination between wild-type and mutant alleles (G12R, G12D), thus improving the interpretability of dPCR results [194]. Yixin Fu et al. introduced a ligation-initiated LAMP strategy capable of detecting mutations at attomolar (aM) sensitivity, even with abundant wild-type DNA, suggesting future ultra-sensitive, point-of-care applications [195].

More recently, Mansour et al. demonstrated the feasibility of ddPCR for KRAS testing in cytology and EUS-FNA samples from pancreatic lesions, where DNA yield is limited, highlighting ddPCR’s robustness for low-input clinical materials [196]. Shimane et al. also validated a PNA-directed PCR clamping assay in peritoneal washing cytology of pancreatic ductal adenocarcinoma (PDAC), demonstrating that clamp-based PCR enables mutation detection even in sparse cytology fluids [197].

A pivotal clinical consideration is assay mutation coverage. The FDA-approved therascreen KRAS assay, a companion diagnostic widely employed for colorectal and lung cancers, omits codon 61 mutation detection. Given that approximately 5% of NSCLC KRAS mutations localize at Q61 codons, this gap poses a significant risk of patient misclassification and loss of access to appropriate EGFR antibody therapies or enrollment in emerging KRAS-targeted trials [73,183]. Comprehensive platforms such as expanded NGS panels or emerging multiplexed assays covering rarer mutations (e.g., codon 117, 146) mitigate this risk and are therefore preferred in contexts demanding exhaustive mutational profiling [185].

Emerging ultra-sensitive approaches, including digital PCR and novel blocker-based enrichment strategies, have revolutionized liquid biopsy applications. With allele detection sensitivity down to 0.01%, these platforms are instrumental in ctDNA analysis for minimal residual disease monitoring, early relapse detection, and longitudinal assessment of therapeutic resistance [198]. Ye et al.’s 2020 meta-analysis confirmed the clinical validity of digital PCR in plasma for colorectal cancer KRAS mutation detection, marking a paradigm shift towards non-invasive, dynamic tumor genotyping [198]. Jun et al. further showed that ddPCR achieves 100% sensitivity and specificity for low-frequency KRAS mutations (<0.01%), outperforming Sanger and clamping assays, though further multi-center validation is needed prior to widespread clinical adoption [130].

Expanding on this, Addamo-De Nard et al. introduced a novel drop-off ddPCR assay targeting exon 2 mutations, which achieved excellent specificity and sensitivity in cfDNA from colorectal cancer patients, showing promise for rapid integration into clinical liquid biopsy pipelines [163].

Additional innovations leverage biosensor technologies, droplet microfluidics, and lab-on-chip assays integrating isothermal amplification (LAMP) or molecular clamping (PNA/XNA). For instance, the fluorescence-based LBDA method successfully analyzes 81 KRAS hotspots rapidly with high sensitivity and specificity, promising scalable, cost-effective alternatives to NGS for broad hotspot detection in routine practice. Furthermore, machine learning-assisted predictive models have recently demonstrated high prognostic and predictive power based on mutation signatures and imaging data, suggesting integration potential for morphology-driven mutation prediction pending broader validation [162,199].

Regulatory landscapes introduce further complexity. FDA-IVD and CE-IVD approvals confer confidence in assay reliability, yet differences in approved mutation panels and platforms remain. Clinicians and molecular diagnostics laboratories must critically assess assay regulatory status alongside performance metrics, ensuring alignment with intended clinical uses and population mutation spectra. Notably, the lack of codon 61 inclusion in FDA-cleared assays mandates supplementary testing strategies or reflexive NGS to avoid undertreatment [183]. Table 4 summarizes companion diagnostic devices/in vitro and imaging tools approved or cleared by the FDA.

In practice, no single methodology suffices universally. Instead, a multi-modal, context-dependent testing algorithm accounting for clinical indication, specimen type, mutation prevalence, and turnaround times optimizes outcomes. Orthogonal validation of equivocal cases using complementary assays fortifies diagnostic accuracy and reduces false negatives, enhancing patient stratification with downstream therapeutic consequences [183,185,191].

Although numerous KRAS detection platforms have been developed, their performance in real-world clinical settings remains heterogeneous. Many assays report excellent analytical sensitivity under controlled laboratory conditions, yet their ability to detect low-allele-frequency variants in highly heterogeneous tumors is still limited. In particular, PCR-based techniques are highly dependent on primer design and may generate false positives, whereas sequencing-based approaches often require high depth, sophisticated bioinformatics, and significant cost.

Another unresolved challenge is the lack of universal standardization across laboratories. Variability in sample preparation, amplification protocols, and variant-calling pipelines can lead to inconsistent results, complicating inter-laboratory comparison and potentially influencing therapeutic decisions.

In conclusion, the rapidly evolving KRAS mutation detection armamentarium offers an expanding toolkit to realize precision oncology. Careful method selection, based on sensitivity, coverage, sample-type suitability, regulatory clearance, and cost-effectiveness, remains paramount to clinical success. Integration of ultra-sensitive liquid biopsies, innovative multiplex platforms, and computational prediction promises to further refine patient care pathways and heralds a future of personalized, dynamic cancer management.

## 11. Future Perspectives

The future of personalized medicine holds great promise, and detecting KRAS mutations is set to play a pivotal role in shaping this landscape. KRAS mutations are prevalent in various cancers, making them essential biomarkers for tailoring treatment strategies. Emerging technological advancements are revolutionizing how we detect and analyze these mutations, paving the way for more effective and targeted therapies. One of the most significant developments is transitioning from traditional methods to highly sophisticated instruments, such as next-generation sequencing (NGS) and digital PCR, which offer unparalleled sensitivity and specificity. These advanced techniques enable the detection of KRAS mutations with greater precision and facilitate the identification of rare variants that were previously challenging to capture. This finer granularity in mutation profiling allows clinicians to make more informed decisions, selecting therapies that are more likely to succeed and minimizing the risk of adverse effects. Moreover, miniaturization technologies like lab-on-disk, lab-on-chip, and microfluidics drive the field toward more efficient and cost-effective mutation detection. Lab-on-disk platforms, for instance, can automate sample preparation, reducing the potential for human error and streamlining the workflow.

On the other hand, lab-on-chip devices enable the integration of various analytical processes into a single compact system, making it feasible to perform multiplexed KRAS mutation analysis rapidly and in a point-of-care setting. Thanks to manipulating minute volumes of fluids, microfluidics facilitates high-throughput screening and single-cell analysis, opening doors to a deeper understanding of tumor heterogeneity and treatment resistance mechanisms. As we look to the future, these technological advancements will not only enhance the accuracy and efficiency of KRAS mutation detection but will also drive down costs, making personalized medicine more accessible to a broader patient population. With the advent of liquid biopsies, which can detect KRAS mutations from a simple blood sample, monitoring disease progression and treatment response will become less invasive and more convenient for patients. This shift towards non-invasive diagnostics holds the potential to revolutionize cancer care, allowing for real-time monitoring and prompt adjustments to treatment plans.

Despite remarkable scientific progress, major gaps remain in our understanding of KRAS-driven oncogenesis and therapeutic vulnerabilities. The field still lacks biomarkers that robustly predict the response to KRAS inhibitors, and the optimal sequencing of KRAS-targeted therapies with chemotherapy, immunotherapy, or radiotherapy remains unclear.

Additionally, the rapid expansion of KRAS G12D and pan-KRAS inhibitors raises important concerns regarding tumor adaptation, long-term toxicity, and potential selective pressures that may foster even more aggressive subclones. These issues warrant careful investigation in both preclinical and clinical settings.

Furthermore, integrating artificial intelligence (AI) and machine learning into KRAS mutation detection workflows will enable rapid data analysis and interpretation. AI algorithms can identify complex mutation patterns and predict responses to targeted therapies, guiding clinicians in selecting the most effective treatment options for individual patients. This synergy between advanced technology and computational power will further refine the practice of personalized medicine, improve patient outcomes and reduce the burden of trial-and-error approaches.

## 12. Conclusions

In conclusion, the future of personalized medicine hinges on the accurate and efficient detection of KRAS mutations. Ongoing technological advancements in instruments, lab-on-disk, lab-on-chip, and microfluidics are set to transform the landscape of cancer diagnosis and treatment. These innovations promise greater precision in mutation profiling as well as increased accessibility, affordability, and patient convenience. As we continue to harness the power of these technologies and integrate AI-driven insights, personalized medicine will undoubtedly play an increasingly vital role in fighting cancer and other diseases, ushering in an era of more effective and tailored therapies.

## Figures and Tables

**Table 1:** Summary of the major RASopathies, their associated genes, and their key clinical features.

Disorder	Main Causative Genes	Key Clinical Features	References
Neurofibromatosis type 1 (NF1)	NF1	Cafأ-au-lait spots, neurofibromas, learning issues	[21,22]
Noonan syndrome (NS)	PTPN11, SOS1, RAF1, KRAS, MRAS, RIT1	Short stature, heart defects, distinctive facies	[23]
Cardiofaciocutaneous (CFC)	BRAF, MEK1, MEK2, KRAS	Facial anomalies, intellectual disability, ectodermal defects	[24,25]
Costello syndrome	HRAS	Growth delay, cardiomyopathy, tumor predisposition	[26,27]
Legius syndrome	SPRED1	Cafأ-au-lait spots, mild learning issues	[28]
Other rare forms	PTPN11/RAF1 (NSML), RASA1/EPHB4 (CM-AVM)	Variable features depending on syndrome	[20]

**Table 2:** Key KRAS inhibitors and upstream targeting strategies.

Target	Drug	Mechanism/Class	Clinical Status	References
KRAS (direct)	Sotorasib	KRAS G12C covalent inhibitor	FDA approved (NSCLC)	[46,48]
	Adagrasib	KRAS G12C covalent inhibitor	FDA approved (NSCLC)	[47,50]
	ARS-3248	KRAS G12C inhibitor	Phase I	[51]
	MRTX1133	KRAS G12D selective inhibitor	Preclinical/early clinical	[59]
	RMC-6236	Pan-KRAS multi-mutant inhibitor	Phase I/II	[60]
	BI-2493/BI-2865	Pan-KRAS inhibitor targeting multiple KRAS variants	Preclinical	[61,62]
SOS1	BI-3406	SOS1–KRAS interaction inhibitor	Phase I	[52,53]
SHP2	RMC-4630	SHP2 allosteric inhibitor	Phase I/II	[53]
	TNO155	SHP2 inhibitor	Phase I	[54]
Farnesyltransferase	Tipifarnib	FTI (membrane localization)	Phase II/III	[55,56]
Other emerging strategies	siRNA, degraders, vaccines	Indirect KRAS pathway targeting	Preclinical/early clinical	[46]

**Table 3:** Comparative overview of KRAS mutation detection methods integrating technical, clinical, and regulatory aspects.

Method/Technology	KRAS Codons/Exons Covered	Typical Sample Types	Sensitivity (LoD, %)	Turnaround Time	Main Cancer Applications	Clinical Applications	Key Strengths	Key Limitations	Regulatory/Commercial Kits	Validation Data	References
Sanger Sequencing	Exons 2–4 (12, 13, 61, 146)	FFPE, biopsy, cytology	10–20	1–2 d	CRC, PDAC, NSCLC (historical)	Mutation confirmation, diagnostic classification	Detects novel/rare variants; gold standard	Low sensitivity; poor for cfDNA	Mostly RUO; pathology labs	~95% specificity; poor at <15% VAF	[78,89,90,91,92,93,94]
Pyrosequencing	Exons 2–3 (hotspots)	FFPE, cytology, plasma cfDNA	2–5	<1 d	CRC, NSCLC, PDAC	Routine hotspot profiling; cfDNA screening	Quantitative, rapid	Panel-limited; affected by homopolymers	QIAGEN PyroMark (CE-IVD)	90–95% sensitivity; >95% concordance with NGS	[95,96,97,98,99,100,101,102]
NGS	Exons 2–4; rare/novel variants	FFPE, plasma cfDNA	0.2–5 (≤0.1 ultra-deep)	3–7 d	CRC, PDAC, NSCLC, biliary, endometrial	Comprehensive profiling; detection of co-mutations; trial enrollment	Broad coverage; detects rare variants	High cost; longer TAT	Praxis Extended RAS Panel (FDA), FoundationOne^®^ CDx	≥95% concordance; detects variants missed by PCR	[103,104,105,106,107,108,109,110,111]
Allele-Specific PCR (ARMS/CAST/Scorpion)	Codons 12, 13, 59, 61, 117, 146	FFPE, plasma	0.1–1	<1 d	CRC, NSCLC	FDA-approved companion diagnostics for anti-EGFR therapy	High sensitivity; rapid; regulatory approved	Only detects known variants	Qiagen therascreen^®^ (FDA), EntroGen ARMS	FDA-CDx approved for cetuximab/panitumumab	[112,113,114,115,116,117,118,119,120,121,122,123,124,125,126,127,128]
Digital PCR (ddPCR, cdPCR)	Hotspots; customizable	cfDNA, FFPE, plasma	0.01–0.1	4–6 h	CRC, PDAC (MRD), NSCLC (G12C)	cfDNA MRD monitoring; resistance tracking	Ultra-sensitive; quantitative	Expensive equipment; targeted panels only	Bio-Rad ddPCR kits (RUO); OncoBEAM^®^ (CE-IVD)	≥99% specificity; >95% concordance with tissue	[129,130,131,132,133,163,164]
High-Resolution Melt Analysis (HRMA)	Exons 2–3, 4	FFPE, plasma, biopsy	3–10	<4 h	CRC, PDAC	Rapid triage, screening	Fast; cost-effective	Needs confirmatory assay; moderate sensitivity	cobas^®^ KRAS TaqMelt (Roche, CE-IVD)	85–90% concordance with sequencing	[165,166,167,168,169,170,171]
COLD-PCR	Codons 12, 13	Plasma, FFPE	0.1–1	~6 h	CRC	Enrichment of rare alleles for downstream sequencing	Improves sensitivity; recovers low-frequency mutations	Workflow complex; RUO	No IVD kits	Detects <1% VAF in CRC cfDNA	[134,135,136,137]
LAMP/PNA/XNA Clamp	Codons 12, 13 (custom)	FFPE, plasma, cytology	0.1–2	<2 h	CRC, NSCLC (experimental)	Rapid hotspot detection, POC	Isothermal, fast, portable	Few validated kits; risk of false positives	PNAClamp™ KRAS (CE-IVD, Korea)	80–90% concordance vs. PCR	[138,139,140,141,142,143,144,145,146,147,172]
MassARRAY (MALDI-TOF)	Up to 40 codons	FFPE, plasma	1–5	1–2 d	CRC, PDAC, NSCLC	Multiplex hotspot profiling	High throughput, quantitative	Expensive equipment; central labs	Agena iPLEX^®^ HS Colon Panel (CE-IVD/RUO)	>95% specificity; high concordance with sequencing	[148,149,150,151]
DNA Microarray/xMAP	Dozens of codons	FFPE, plasma	~1	1–2 d	CRC, NSCLC	High-throughput multiplex mutation screening	Multiplex; automation	Lower sensitivity vs. PCR/NGS	Randox RAS Array (IVD), Luminex xMAP RUO	85–90% sensitivity; moderate concordance	[148,152,153]
SMAP-2 (Smart Amplification v2)	Codons 12, 13	Plasma, FFPE	~1	30–60 min	CRC (experimental)	Rapid hotspot detection	Isothermal; no thermocycler	Limited validation; RUO	Experimental	≥90% sensitivity; small panels	[173,174,175,176]
Padlock Probes and RCA	Codons 12, 13, 61	Plasma cfDNA, RNA, FFPE	≤1	6–8 h	CRC, NSCLC (experimental)	Single-molecule detection	Very high sensitivity	Complex workflow; RUO	None; academic	Detects sub-1% VAF in CRC cfDNA	[122,145,146,147,156,177,178,179,180,181]
ISAD (Isothermal Solid-Phase Amplification/Detection)	Codons 12, 13	Plasma, CRC tissue	~1	<30 min	CRC	Rapid label-free hotspot detection	Ultra-fast (<30 min)	Limited to few hotspots; experimental	Prototype chips only	>90% sensitivity in CRC	[154,155,156,157]
Lab-on-Chip/Microfluidics	Customizable	Plasma, cfDNA, FNA	≤1	1–2 h	CRC, PDAC (liquid biopsy)	POC, integrated testing	Automated; scalable	RUO; not widely validated	Hybcell (RUO), In-Check™	~92% concordance vs. ddPCR	[158,159,160,161]
LBDA (Long Block Displacement Amplification)	81 hotspots	FFPE, synthetic DNA	0.08 VAF	<3 h	CRC (validation); PDAC/NSCLC (potential)	High-throughput KRAS hotspot panel	Ultra-sensitive; low cost	New; limited validation	Experimental	88% sens., 100% spec. vs. NGS	[162]

**Table 4:** Companion diagnostic devices (also known as in vitro and imaging tools) cleared or approved by the Food Drug Administration (FDA) (https://www.fda.gov/medical-devices/in-vitro-diagnostics/list-cleared-or-approved-companion-diagnostic-devices-in-vitro-and-imaging-tools (accessed on 3 May 2025)).

Manufacturer (Diagnostic Name)	Indication-Sample Type	Drug Trade Name (Generic) NDA/BLA	Biomarker(s)	Biomarker(s) (Details)	PMA/510(k)/513(f)(2)/HDE (Approval/Clearance/Grant Date)
Agilent Resolution ctDx FIRST assay (Resolution Bioscience, Inc.)	Non-Small Cell Lung Cancer (NSCLC)-Plasma	Krazati (adagrasib) NDA 216340	KRAS	KRAS G12C	P210040 (12 December 2022)
cobas KRAS Mutation Test (Roche Molecular Systems, Inc.)	Colorectal Cancer-Tissue	Erbitux (cetuximab) BLA 125084		Mutations in codons 12 and 13 of the KRAS gene	P140023 (7 May 2015)
cobas KRAS Mutation Test (Roche Molecular Systems, Inc.)	Colorectal Cancer-Tissue	Vectibix (panitumumab) BLA 125147	KRAS	Mutations in codons 12 and 13 of the KRAS gene	P140023 (7 May 2015)
FoundationOne CDx (Foundation Medicine, Inc.)	Colorectal Cancer-Tissue	Erbitux (cetuximab) BLA 125084	KRAS	KRAS wild type (absence of mutations in codons 12 and 13)	P170019 (30 November 2017)
FoundationOne CDx (Foundation Medicine, Inc.)	Colorectal Cancer-Tissue	Vectibix (panitumumab) BLA 125147	KRAS	KRAS wild-type (absence of mutations in exons 2, 3, and 4) and NRAS wild-type (absence of mutations in exons 2, 3, and 4)	P170019 (30 November 2017)
Guardant360 CDx (Guardant Health, Inc.)	Non-Small Cell Lung Cancer (NSCLC)-Plasma	Lumakras (sotorasib) NDA 214665	KRAS and NRAS	G12C	P200010/S002 (28 May 2021)
ONCO/Reveal Dx Lung & Colon Cancer Assay (O/RDx-LCCA) (Pillar Biosciences, Inc.)	Colorectal Cancer-Tissue	Erbitux (cetuximab) BLA 125084	KRAS	KRAS wild type (absence of mutations in codons 12 and 13)	P200011 (30 July 2021)
ONCO/Reveal Dx Lung & Colon Cancer Assay (O/RDx-LCCA) (Pillar Biosciences, Inc.)	Colorectal Cancer-Tissue	Vectibix (panitumumab) BLA 125147	KRAS	KRAS wild type (absence of mutations in codons 12 and 13)	P200011 (30 July 2021)
Praxis Extended RAS Panel (Illumina, Inc.)	Colorectal Cancer-Tissue	Vectibix (panitumumab) BLA 125147	KRAS	KRAS wild-type (absence of mutations in exons 2, 3, and 4) and NRAS wild-type (absence of mutations in exons 2, 3, and 4)	P160038 (29 June 2017)
therascreen KRAS RGQ PCR Kit (Qiagen Manchester, Ltd.)	Colorectal Cancer-Tissue	Vectibix (panitumumab) BLA 125147	KRAS and NRAS	G12A, G12D, G12R, G12C, G12S, G12V, G13D	P110027 (23 May 2014)
therascreen KRAS RGQ PCR Kit (Qiagen Manchester, Ltd.)	Non-Small Cell Lung Cancer (NSCLC)-Tissue	Lumakras (sotorasib) NDA 214665	KRAS	G12C	P110027/S012 (28 May 2021)
therascreen KRAS RGQ PCR Kit (Qiagen Manchester, Ltd.)	Colorectal Cancer-Tissue	Erbitux (cetuximab) BLA 125084	KRAS	G12A, G12D, G12R, G12C, G12S, G12V, G13D	P110030 (6 July 2012)
therascreen KRAS RGQ PCR Kit (Qiagen Manchester, Ltd.)	Colorectal Cancer-Tissue	Erbitux (cetuximab) BLA 125084	KRAS	KRAS wild type (absence of mutations in codons 12 and 13)	P110027/S013 (2 December 2022)
therascreen KRAS RGQ PCR Kit (Qiagen Manchester, Ltd.)	Non-Small Cell Lung Cancer (NSCLC)-Tissue	Krazati (adagrasib) NDA 216340	KRAS	KRAS G12C	P110027/S013 (2 December 2022)

## Abbreviations

The following abbreviations are used in this manuscript:

Signaling molecules and oncogenes:	
AKT	Protein Kinase B
APC	Adenomatous Polyposis Coli
BRAF	B-Raf Proto-Oncogene
EGFR	Epidermal Growth Factor Receptor
GEFs	Guanine Nucleotide Exchange Factors
GAP	GTPase-Activating Protein
GDP	Guanosine Diphosphate
GTP	Guanosine Triphosphate
GTPase	Guanosine Triphosphatase
HGF	Hepatocyte Growth Factor
HRAS	Harvey Rat Sarcoma Viral Oncogene Homolog
KRAS	Kirsten Rat Sarcoma Viral Oncogene Homolog
NRAS	Neuroblastoma RAS Viral Oncogene Homolog
MAPK	Mitogen-Activated Protein Kinase
PI3K	Phosphoinositide 3-Kinase
PIK3CA	Phosphatidylinositol-4,5-Bisphosphate 3-Kinase Catalytic Subunit Alpha
SHP2	Src Homology Region-2 Containing Protein Tyrosine Phosphatase
SOS1	Son of Sevenless-1
Mutations and amino acid substitution codes:	
CAAX	Cysteine–Aliphatic–Any Amino Acid motif
L858R	Leucine-to-Arginine substitution at EGFR codon 858
T790M	Threonine-to-Methionine substitution at EGFR codon 790
Diagnostic biomarkers:	
CA19-9	Cancer Antigen 19-9
cfDNA	Cell-Free DNA
ctDNA	Circulating Tumor DNA
FFPE	Formalin-Fixed Paraffin-Embedded
FFPET	Formalin-Fixed Paraffin-Embedded Tissue
FNA	Fine-Needle Aspiration
IVD	In Vitro Diagnostic
*PCR-based and isothermal amplification methods:*	
AS-ARMS PCR	Allele-Specific Amplification Refractory Mutation System PCR
AS-PCR	Allele-Specific PCR
AS-LAMP	Allele-Specific or Asymmetric Loop-Mediated Isothermal Amplification
AS-SSCP	Allele-Specific Single-Strand Conformational Polymorphism
CAST-PCR	Competitive Allele-Specific TaqMan PCR
ddPCR	Droplet Digital PCR
dPCR	Digital PCR
EAB-qPCR	Enhanced Asymmetric Blocked qPCR
HRMA	High-Resolution Melting Analysis
LB	Loop-Back Primer
LAMP	Loop-Mediated Isothermal Amplification
PCqPCR	Peptide Nucleic Acid Clamp Real-Time PCR
PCR	Polymerase Chain Reaction
qPCR	Quantitative PCR
RFLP	Restriction Fragment Length Polymorphism
RPA	Recombinase Polymerase Amplification
SMAP-2	Smart Amplification Process v2
SSCP	Single-Strand Conformational Polymorphism
TaqMelt^®^	High-Resolution Taq-Based Melt Assay
Tc	Critical Denaturation Temperature
Tm	Melting Temperature
Next-generation and high-throughput platforms:	
NGS	Next-Generation Sequencing
MALDI-TOF	Matrix-Assisted Laser Desorption/Ionization Time-of-Flight
MassARRAY	Mass Spectrometry-Based Genotyping System
LOC	Lab-On-Chip
MEMS	Microelectromechanical Systems
MILLIPLEX	Multiplex Assay Technology
SMR	Silicon Microring Resonator
xMAP	Multi-Analyte Profiling
Synthetic probes and clamps:	
BODIPY	Boron-Dipyrromethene
FAM	Fluorescein Amidite
PNA	Peptide Nucleic Acid
XNA	Xeno-Nucleic Acid
QClamp^®^	XNA-Based Mutation Test
Clinical assays, kits, or commercial platforms:	
AMG-510	Sotorasib
MRTX849	Adagrasib
cobas^®^	Roche qPCR Mutation Test
PSS	Plasma-SeqSensei™
iPLEX^®^ HS	High-Sensitivity iPLEX Panel
OncoBEAM^®^	Digital PCR-Based BEAMing Assay
Disease names and clinical terms:	
CRC	Colorectal Cancer
mCRC	Metastatic Colorectal Cancer
NSCLC	Non-Small Cell Lung Cancer
CFC	Cardiofaciocutaneous Syndrome
NS	Noonan Syndrome
NF1	Neurofibromatosis Type 1
Other technical terms:	
Ct	Cycle Threshold
CV%	Coefficient of Variation Percentage
DNA	Deoxyribonucleic Acid
RNA	Ribonucleic Acid
ECL	Electrochemiluminescence
FDA	Food and Drug Administration
RUO	Research Use Only
